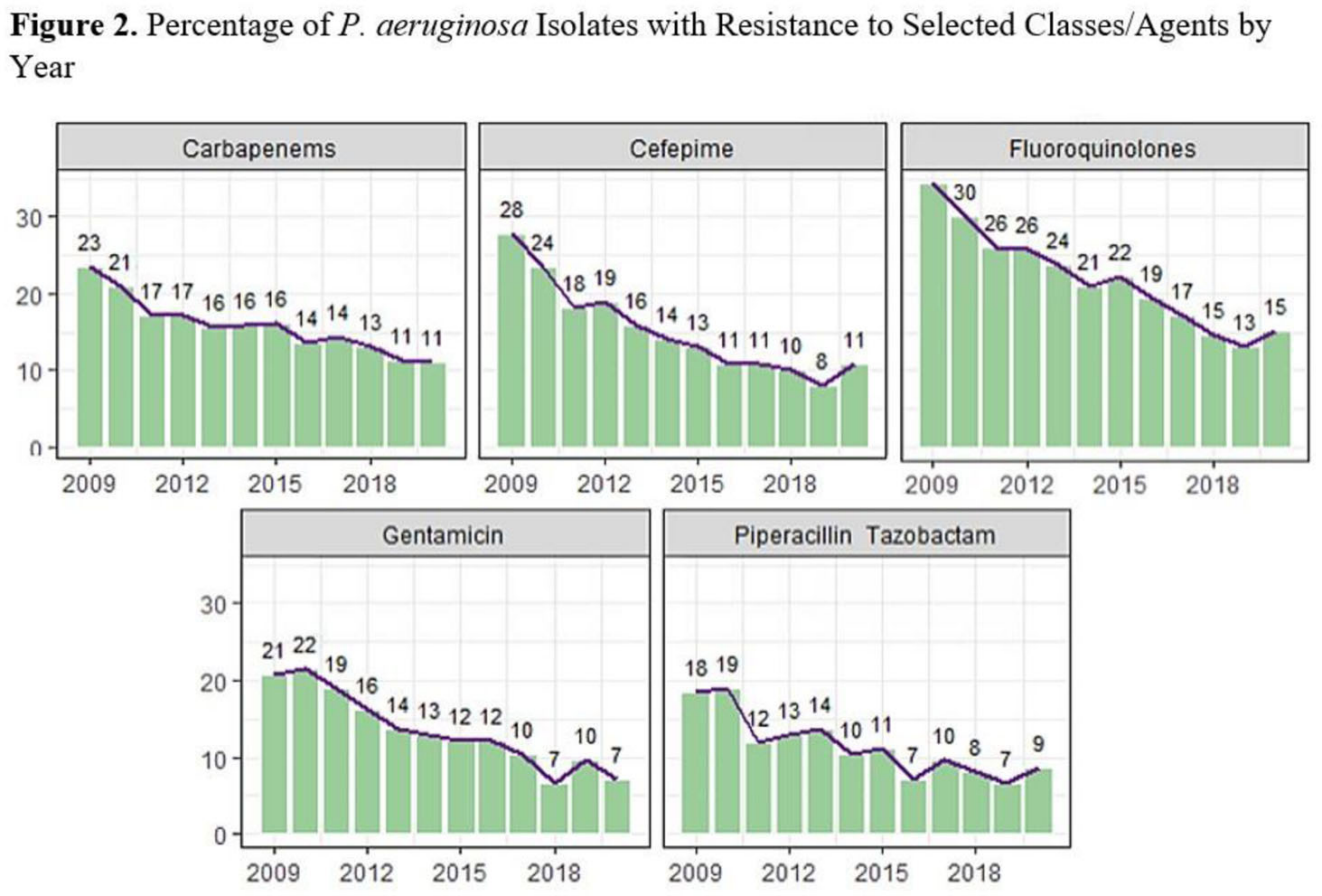# *Pseudomonas aeruginosa* bacteremia mortality and resistance trends in the Veterans’ Health Administration (VHA) system

**DOI:** 10.1017/ash.2022.155

**Published:** 2022-05-16

**Authors:** Leila Hojat, Brigid Wilson, Federico Perez, Robert Bonomo

## Abstract

**Background:** *Pseudomonas aeruginosa* is an important pathogen in the hospital setting; it has the ability to cause severe disease and a high mortality rate. Its increasing ability to elude even novel antimicrobial mechanisms of action is a significant cause for concern. More effective treatment options and increasing understanding of this pathogen likely effect *P. aeruginosa* incidence and severity; however, longer-term studies are lacking. The Veterans’ Health Administration (VHA) population is a socially, demographically, and medically distinct entity, representing a rich source of data for studying contributing factors to *P. aeruginosa* infection and mortality. We sought to identify the system-wide case count and mortality rate of *P. aeruginosa* bacteremia and the rate of resistance to antipseudomonal agents over the course of several years. We described trends observed over the study period. **Methods:** We utilized the nationwide VHA database to identify all inpatients with a positive blood culture for *P. aeruginosa* treated between January 1, 2009, and December 31, 2020. We identified the annual count of bacteremia cases and associated 30-day mortality rate. Additionally, we determined rates of resistance to antipseudomonal agents. **Results:** In total, 7,480 cases of *P. aeruginosa* bacteremia were identified. The total case count of *P. aeruginosa* bacteremia decreased from 774 in 2009 to 519 in 2014, then remained relatively stable. The 30-day mortality rate decreased from 26.5 in 2009 to 19.3 in 2019, but this rate increased to 23.6 in 2020 (Fig. 1). The fluoroquinolone class had the highest resistance rate at 23%, followed by ceftazidime, cefepime, and the carbapenem class with rates of ~15%–16%. All classes were noted to have decreased resistance over time (Fig. 2). **Conclusions:** Occurrences, mortality rate, and associated resistance of *P. aeruginosa* bacteremia across the VHA system generally decreased during the study period. Potential explanations for these observations include improved infection control measures, more effective therapeutic agents, and enhanced antimicrobial stewardship efforts. The increased mortality in 2020 could be related to concomitant COVID-19 or the result of delayed medical care in the pandemic setting. Limitations of this study include inability to identify causative factors for observed trends and potential variability between labs affecting the rates of observed resistance. Additionally, VHA data may not be representative of entire adult population. Future studies could explore the relationship between *P. aeruginosa* bacteremia and infection prevention and antimicrobial stewardship efforts and could describe associations between *P. aeruginosa* and COVID-19 and identify risk factors associated with *P. aeruginosa* bacteremia and mortality.

**Funding:** None

**Disclosures:** None

**Figure:**
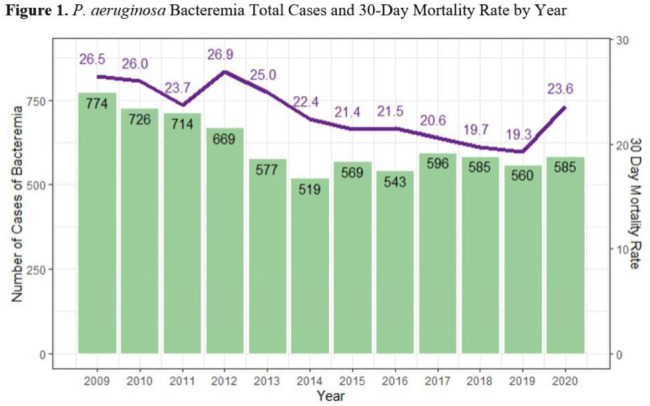

**Figure:**